# Leadership and post-traumatic stress disorder: are soldiers’ perceptions of organizational justice during deployment protective?

**DOI:** 10.1080/20008198.2018.1449558

**Published:** 2018-04-04

**Authors:** Andreas F. Elrond, Annie Høgh, Søren B. Andersen

**Affiliations:** a Research and Knowledge Centre, The Danish Veteran Centre, Ringsted, Denmark; b Department of Psychology, University of Copenhagen, København K, Denmark

**Keywords:** Traumatic stress, military deployment, perception of leadership, procedural justice, interactional justice, alarm system perspective, estrés traumático, despliegue militar, percepción del liderazgo, justicia procedimental, justicia interaccional, perspectiva del sistema de alarma, 创伤应激, 入伍, 领导力感知, 过程公平, 互动公平, 预警系统观点, • Soldiers’ perceptions of leadership in relation to military deployment can be understood through theories of organizational justice. • Procedural and interactional justice before and during military deployment to a combat zone relates to postdeployment PTSD. • Soldiers may be using perceptions of leadership as a proxy for judgements of safety in a combat zone. • Modifying factors that lead to perceptions of procedural and interactional justice may protect against PTSD.

## Abstract

**Background**: Soldiers’ perception of leadership during military deployment has gained research attention as a potentially modifiable factor to buffer against the development of postdeployment post-traumatic stress disorder (PTSD). Within nonmilitary research, the organizational justice (OJ) framework, i.e. distributive justice, procedural justice (PJ) and interactional justice (IJ), has been found to relate to mental health outcomes. Aspects of OJ may, therefore, be protective against PTSD.

**Objectives**: We examined the prospective relationship between aspects of OJ, namely the perceptions of PJ and IJ by subordinate soldiers without leadership obligations in relationship to immediate superiors and PTSD.

**Method**: Participants were soldiers (*n = *245) deployed to Helmand Province in Afghanistan in 2009. Logistic regression procedures were used. The primary analysis measured PTSD cases using the Structured Clinical Interview for DSM-IV-TR Axis-I Disorder (SCID) 2½ years after homecoming. PJ/IJ was measured during deployment with a 6-item composite measure ranging from 0 to 12. Supplementary primary analyses were performed with PJ/IJ measured before and immediately after deployment. A secondary PJ/IJ analysis also tested against four postdeployment measures with the Post-Traumatic Stress Disorder Checklist Civilian (PCL-C) dichotomized at screening symptom levels.

**Results**: Higher levels of perceived PJ/IJ for soldiers without leadership obligations during deployment had a prospective relation (OR = 0.86, 95% CI = 0.75–0.98) with PTSD on the SCID 2½ years after homecoming after adjustment for factors including predeployment PTSD symptoms, trauma and combat exposure, and state affectivity. Similar results were found by measuring PJ/IJ before (OR = 0.83, 95% CI = 0.71–0.95) but not immediately after homecoming (OR = 0.97, 95% CI = 0.85–1.11). A relationship with PTSD symptoms at the screening level at the four measurements of PCL-C was found, but only when predeployment PTSD symptoms were not controlled for.

**Conclusions**: These results suggest that PJ/IJ exercised by superiors in relation to military deployments may protect subordinate soldiers against the development of postdeployment PTSD.

## Abstract (es)

**Planteamiento:** La percepción del liderazgo de los soldados durante un despliegue militar se ha ganado la atención de la investigación como factor potencialmente modificable para amortiguar el desarrollo del trastorno por estrés postraumático (TEPT) después de un despliegue militar. Dentro de la investigación no militar, se ha encontrado que el marco de justicia organizacional (JO) –es decir, justicia distributiva, justicia procedimental (JP) y justicia interaccional (JI)– está relacionado con resultados de salud mental. Algunos aspectos de la JO pueden, por lo tanto, proteger contra el TEPT.

**Objetivos:** Examinamos la relación prospectiva entre los aspectos de la JO, es decir, las percepciones de JP y JI de los soldados subordinados sin obligaciones de liderazgo en relación a sus superiores inmediatos y al TEPT. Método: Los participantes fueron soldados (*n = *245) desplegados en la provincia de Helmand en Afganistán en 2009. Se usaron procedimientos de regresión logística. El análisis principal midió los casos de TEPT mediante la Entrevista Clínica Estructurada para los trastorno del Eje I del DSM-IV-TR (SCID, por sus siglas en inglés) dos años y medio después del regreso a casa. Se midieron la JP y la JI durante el despliegue con una medida compuesta de seis elementos que van de 0 a 12. Se realizaron análisis primarios adicionales, midiendo la JP y la JI antes e inmediatamente después del despliegue. Un análisis secundario de la JP y la JI también se comparó con cuatro medidas posteriores al despliegue con la Lista de verificación del trastorno por estrés postraumático civil (PCL-C, por sus siglas en inglés) dicotomizada para los niveles de síntomas en la selección.

**Resultados:** Niveles más altos de percepción de JP / JI en soldados sin obligaciones de liderazgo durante el despliegue tuvieron una relación prospectiva (OR = 0.86, 95% -IC = 0.75–0.98) con el TEPT en el SCID 2½ años después del regreso al hogar por factores que incluían síntomas de TEPT previos al despliegue, exposición al trauma y al combate, y afectividad de estado. Se encontraron resultados similares midiendo la JP y la JI antes (OR = 0,83; IC del 95% = 0,71–0,95) pero no inmediatamente después de la vuelta al hogar (OR = 0,97; IC del 95% = 0,85–1,11). Se encontró una relación con los síntomas de TEPT en el nivel de detección en las cuatro medidas del PCL-C, pero solo cuando no se controlaron los síntomas del TEPT antes del despliegue.

**Conclusiones:** Estos resultados sugieren que la JP y la JI ejercidas por los superiores en relación con los despliegues militares puede proteger a los soldados subordinados de desarrollar un TEPT posterior al despliegue.

## Abstract (zh)

**背景：**研究者开始关注，在军队服役过程中，士兵对领导力的主观感受可能是缓冲服役后创伤后应激障碍（PTSD）的一个可调控的因素。在非军队研究中，组织公平（OJ）框架——分配公平、过程公平（PJ）和互动公平（IJ）——被发现和心理健康有关。因此，OJ 的一些方面可能是应对PTSD 的保护因素。

**目标：**我们考察了组织公平（OJ）各方面的前瞻性关系：即没有领导责任的下属士兵对直接长官的 PJ 和 IJ的主观感受及其 PTSD 之间的关系。

**方法：**245名被试在2009年应征到阿富汗的赫尔曼德省。使用 Logistic 回归方法分析数据。主要分析使用 《DSM-IV-TR 轴I障碍的临床结构访谈》（SCID）在归国2.5年后测量了 PTSD。使用6道复合题来测量服役中的PJ/IJ（从0到12分），附加分析了服役前和服役后的PJ/IJ 。次级PJ/IJ 分析中考查了其对PTSD症状的预测，使用四次服役后的《创伤后应激障碍检查表-国民版》（PCL-C ）筛查症状。

**结果：**无领导责任的服役士兵中，控制了服役前的PTSD、创伤和战争暴露以及情感状态后，发现更高水平的主观PJ/IJ 和归国2.5年后的 PTSD（SCID 测量）有关联性(OR = 0.86, 95% CI = 0.75–0.98) 。相似的结果也出现在归国前的PJ/IJ (OR = 0.83, 95% CI = 0.71–0.95) ，但没有出现在归国短期后的PJ/IJ (OR = 0.97, 95% CI = 0.85–1.11)。只有在服役前PTSD症状没有被控制时，PCL-C的四次测量和PTSD 症状才有关联性。

**结论：**这些结果提示，军队服役中长官的PJ/IJ可能会保护下属士兵在服役后出现PTSD。

## 1. Introduction

The relationship between exposure to traumatic events or combat during military deployments and the risk of developing post-traumatic stress disorder (PTSD) is well established (Berntsen et al., 2012; Iversen et al., 2008; Peterson, Wong, Haynes, Bush, & Schillerstrom, 2010; Xue et al., 2015). However, exposure alone may not fully explain the occurrence of PTSD symptoms, as nontraumatic risk and resilience factors may influence development (Andersen, Karstoft, Bertelsen, & Madsen, 2014; Xue et al., 2015). Identifying modifiable factors that may account for such differences may, therefore, be valuable for preventing the development of both PTSD symptoms and cases of full PTSD.

Military leaders serve a central position in guiding and supporting subordinate soldiers before, during and after deployment (Bartone, 2006; Britt, Wright, & Moore, 2012; Jones et al., 2012; McGurk et al., 2014), and since leaders themselves are aware of such an influence (Adler et al., 2008), leadership may be one such modifiable risk or resilience factor with regards to prevention of PTSD.

That soldiers’ perceptions of leadership are related to PTSD is corroborated by empirical findings, including a recent meta-analysis (Xue et al., 2015, p. 15) and a review (Ramchand, Rudavsky, Grant, Tanielian, & Jaycox, 2015) that found deployment-related stressors, which include having problems with leadership, to be risk factors for the development of PTSD and mental health problems. Several studies further established that subordinate soldiers’ perceptions of leadership during deployment play a pivotal role specifically with respect to the experience of fair and equal treatment, praise, interest and concern (Castro & McGurk, 2007; Du Preez, Sundin, Wessely, & Fear, 2012; Iversen et al., 2008; Jones et al., 2012; McGurk et al., 2014). However, the studies generally lack theoretical explanations for these relationships, which could aid in making perceptions of leadership an actual modifiable factor. One exception in this regard is a study by McGurk et al. (2014), which used the theory of conservation of resources (Hobfoll, 2001) as framework to explain how positive and negative leadership could result in resource gain or loss spirals, which could explain relationships with PTSD. Yet, the use of cross-sectional data restricted the possible interpretation over time.

Drawing on contemporary organizational psychology, the risk and resilience aspects of leadership described above may also be understood through the theories of organizational justice (OJ). OJ refers to employees’ perceptions of being treated fairly and rewarded for their efforts by the organization and the leadership they work for. Early research on OJ focused mainly on the fairness of distribution of rewards under the term ‘distributive justice’, while later research found that the fairness of ‘procedural justice’ (PJ) in organizations is also important for the perception of justice and fairness. Additional theoretical developments also separated the leadership’s enactment of procedures into the term ‘interactional justice’ (IJ) (Bobocel & Gosse, 2015). Nevertheless, PJ and IJ may still be studied in combination (Colquitt, 2012; Colquitt & Shaw, 2005).

The relationships between aspects of OJ and health and well-being outcomes in nonmilitary settings are well established (Ndjaboué, Brisson, & Vézina, 2012; Robbins, Ford, & Tetrick, 2012). However, it has been suggested that reverse causation, i.e. depressive symptoms predicting perceptions of OJ, accounts for at least some of this relation (Lang, Bliese, Lang, & Adler, 2011). Likewise, concurrent affectivity has been related to how OJ is perceived (Barsky & Kaplan, 2007). This calls for the use of longitudinal designs that control for baseline mental health symptoms and concurrent affectivity.

Theoretically, the OJ framework may be especially relevant for the military deployment context, with its multitude of dangers and uncertainties. One line of research into OJ thus suggests that judgements of fair treatment are activated by and help individuals to cope with uncertainties. What has been coined the ‘alarm-system perspective’ suggests that, under alarming conditions, people will try to make sense of uncertainties by judging the fairness of procedures they are engaged in, and they use these perceptions as a substitute for unavailable information about the real risk (van den Bos, 2015). Such cues may then have either a calming effect or give rise to further negative reactions.

Two studies have previously applied the OJ framework to studies in military populations. Olsen, Myrseth, Eidhamar, and Hystad (2012) conducted a cross-sectional validation study of an OJ scale on Norwegian army officers, and Lang et al. (2011) tested the longitudinal direction of the relationship between perceptions of OJ and depressive symptoms in three samples of US soldiers. Neither of the studies tested these relationships in military populations deployed into an active war zone, and no study has to date tested the specific relationship between aspects of OJ during military deployment and postdeployment PTSD.

The aim of this multiwave longitudinal study was to test the hypothesis of a predictive relationship between (a) military personnel’s judgements of procedural and interactional justice (PJ/IJ) during deployment as enacted by their immediate superior and (b) PTSD identified with the Structured Clinical Interview for DSM-IV-TR Axis-I Disorder (SCID) or by self-reported symptoms exceeding a screening cut-off on the Post-Traumatic Stress Disorder Checklist Civilian scale (PCL-C).

## 2. Methods

### 2.1. Participants and procedure

Before, during and after a 6-month deployment to Helmand Province in Afghanistan in 2009, Danish soldiers from the International Security Assistance Force (ISAF) were asked to complete several questionnaires concerning their background and their experiences during and possible reactions to the deployment. The questionnaire data were obtained at a Danish military camp approximately 1–2 months before deployment, at the base or in Afghan airports approximately three months into the deployment, and 1–3 weeks after homecoming, at standard homecoming meetings. Two to three months after their homecoming, the questionnaire data were obtained at military camps or via mail for those now civilians. At 7–8 months, all were contacted via mail, and two cinema tickets were given for their participation. At the time, 2½–3 years after homecoming, soldiers were invited via mail to answer a questionnaire and participate in an SCID, for which they received travel reimbursement and a gift certificate (value ≈ US$80). Participants were continuously informed that the data were being gathered for research purposes only. An overview of the data can be found in Supplemental data Table S1.

The SCID interviews were performed by six graduate psychology students who went through an intensive training programme and a certification course. The interrater reliability of videorecorded SCIDs was tested after one month and showed a .73 for the questions in the SCID module, but there was full agreement on the overall PTSD diagnoses (Karstoft, Andersen, Bertelsen, & Madsen, 2014).

The full sample deployed on the mission consisted of 743 soldiers. Of interest were 458 soldiers who specified having ‘no leadership function’ in the mission area, since the expectation was that soldiers’ own leadership engagement could result in different perceptions of their control over the situation. Of the soldiers without leadership function, 309 participated in the SCID 2½–3 years after their homecoming. Overall, the dropout analysis of participation in the SCID showed no significant differences. The analysis did indicate less participation amongst the younger age groups, but previous research on the present population has not found age to be a predictor for the development of PTSD symptoms (Andersen et al., 2014), therefore we did not take further action. Of the 309 who participated in the SCID, 64 were excluded for not answering all the questions measuring PJ/IJ during deployment or for missing more than one item on the other scales. Furthermore, four did not answer a question on additional deployments or give informed consent for the data used. This left a final study sample of *n = *243 for the primary analysis, whilst varying for supplemental or secondary analyses in accordance with inclusion criteria.

### 2.2. Measures

#### 2.2.1. Post-traumatic stress: SCID, PCL-C

To measure the presence of PTSD we used the SCID (First, Spitzer, Gibbon, & Williams, 2002) approximately 2½ years after soldiers’ homecoming. The SCID provides a dichotomous diagnostic outcome of the presence of PTSD, when criteria are met within the last month before the interview. Using such structured clinical interviews is generally considered ‘the gold standard’ (Karstoft et al., 2014; Richardson, Frueh, & Acierno, 2010). To measure predeployment PTSD symptom levels, we included the 17-item PCL-C (Weathers, Litz, Herman, Huska, & Keane, 1993) that ranges from 17 to 85, as a continuous scale (Cronbach’s α = 0.88). Finally, four postdeployment measures of the PCL-C were used to test for the presence of self-reported PTSD symptoms. Low screening cut-offs for PTSD symptoms (PCL-C > 29) were used to dichotomize the measure, given that there were too few cases exceeding other established screening or clinical cut-offs at three of the four measures (Karstoft et al., 2014).

#### 2.2.2. PJ/IJ

To measure PJ/IJ during deployment, a 6-item composite measure was developed and validated using Rasch procedures, as no other measure had been obtained (Table 1). Description of the scale development can be found in Supplemental data. The PJ/IJ scale had a scoring range of 0–12 (α = 0.91), with higher scores indicating better perception of PJ/IJ.

**Table 1.** Procedural and interactional justice items.

Procedural and interactional justice items
Do you find that your immediate superior has an interest in what goes on amongst the employees?
Do you find that your immediate superior to a reasonable degree takes the individual into account?
Do you find that your immediate superior is treating you in a fair manner?
Do you find that your immediate superior contributes to keeping an open discussion about work-related topics?
Does your immediate superior show you that he/she values the work you are doing?
Do you think that your immediate superior is willing to pass on your wishes and views?

#### 2.2.3. Traumatic stress exposure: Danger/Injury Exposure Scale, Combat Exposure Scale

Potential traumatic experiences were measured with the 10-item Danger/Injury Exposure Scale (DIS; Berntsen et al., 2012) obtained during deployment. The DIS ranges from 10 to 40 (α = 0.83) and targets perceptions of more general traumatic events during military deployment. This scale was chosen since the sample consisted of soldiers performing a range of tasks that could place them in potentially traumatizing situations, not limited to direct combat. To also measure exposure to more direct combat, danger and the deaths of unit members, we further included the 7-item Combat Exposure Scale (CES; Keane et al., 1989), which ranges from 0 to 41 (α = 0.81). Our original study design did intend to use the measure of the DIS obtained at standard homecoming meetings 1–3 weeks after homecoming. However, this measurement point showed signs of inconsistency, i.e. it bore no relationship to future PTSD. We believe this stems from a bias in answers introduced by the situation, possibly in combination with immediate relief, as it was the first official meeting with their comrades (Richardson et al., 2010). Therefore, we used the measures of exposure taken during deployment.

#### 2.2.4. Other factors: Positive And Negative Affect Schedule, age, gender, additional deployment and trauma, Beck Depression Inventory

In all models we included the risk/resilience factors of age 1–2 months before deployment and gender. Furthermore, two measures of positive affectivity (α = 0.87) and negative affectivity (α = 0.80), both ranging from 10 to 50, from the Positive And Negative Affect Schedule (PANAS; Watson, Clark, & Tellegen, 1988), were used to control for concurrent affectivity whilst answering the questionnaire on PJ/IJ. From the Traumatic Life Events Questionnaire (Kubany et al., 2000), a dichotomous indicator of having at least one additional deployment into a war zone and a measure of the number of 16 potentially traumatic events experienced, was introduced to account for experiences between homecoming and participation in the SCID. Finally, the Beck Depression Inventory (Beck, Steer, & Brown, 1996), ranging from 0 to 63, was included in a model to account for baseline depressive symptoms.

### 2.3. Analyses

All data analyses were conducted using R (v. 3.2.3) through RStudio (v. 0.99.491). Descriptive data on the distribution of predictors and additional background information were produced, along with a zero-order correlation between all variables used in the regression models.

For the primary analysis, we used a logistic regression model with incremental adjustment. The ‘basic model’ contained PJ/IJ and predictors related to trauma exposure during this deployment or during additional deployments. The ‘main model’ included the risk/resilience variables age, gender, predeployment PTSD symptoms, the two scales on state affectivity during deployment and the additional trauma exposure before SCID. A ‘predeployment depressive model’ further included baseline depressive symptom levels, to account for the effect of possible reverse causation. Supplementary primary analyses were conducted to test relationships of the PJ/IJ and PANAS measures taken 1–2 months before and 1–3 weeks after with the SCID.

The secondary analysis included the predictors of the ‘main model’ in models with the PCL-C scales dichotomized at the screening cut-off as an outcome, to test the relation to self-reported PTSD symptoms at the four postdeployment measures. The indicators of additional trauma exposure were excluded since these were first measured together with the last PCL-C outcome. In line with previous research conducted on the data (Andersen et al., 2014), we also performed analyses excluding the predeployment PCL-C measure, to account for a possible overadjustment. This decision was further fuelled by results pertaining to the first set of models showing the predeployment PCL-C measure to be a very strong predictor of the dichotomized PCL-C outcome, while it did not relate to the objectively identified SCID outcome in the ‘main model’.

To handle missing data on the predictors, a person-mean imputation was imposed when an answer was missing in each of the scales. Given the more complex scoring of the CES, a population mode imputation was imposed for answers on this measure. For the scale of PJ/IJ, the initial selection ensured that there were no missing data on any items.

## 3. Results

Of the participants (*n = *243), the SCID identified *n = *22 cases and *n = *223 non-cases of PTSD. The distribution of cases by the variables used in the primary analysis and additional background information are presented in Table 2. For an overview, zero-order correlations between all variables can be found in Supplemental data Table S2.

**Table 2.** Descriptive statistics, variables in the main model and additional background information.

PJ/IJ and PANAS measurement	During deployment	
Main study variables based on the SCID outcome^a^	No PTSD (*n = *221)	PTSD (*n = *22)
Predeployment study variables		
Age	29.19 (6.56)	24.95 (5.59)
Female^b^ (%)	7.69	9.09
PCL-C score	22.89 (7.22)	26.55 (9.76)
During deployment study variables		
DIS	18.67 (4.54)	21.77 (5.40)
CES	9.21 (6.28)	12.59 (8.06)
PANAS positive score	28.27 (7.57)	32.64 (7.07)
PANAS negative score	13.71 (4.44)	16.32 (5.78)
PJ/IJ	6.66 (3.65)	4.70 (3.66)
Postdeployment study variables		
Additional deployment before SCID (%)	37.56	45.45
Additional trauma exposure before SCID	1.54 (1.66)	2.91 (1.60)
Additional background information^a,c^	(n ≤ 221)	(n ≤ 22)
Rank at start of deployment		
Privates (%)	90.50	95.45
Sergeants, Officers (%)	8.14	4.55
Unknown, Other (%)	1.36	0.00
Infantry (%)	46.15	59.09
Full duration of deployment	187.95 (21.31)	180.95 (31.38)
Number of previous deployments	1.42 (6.78)	0.50 (0.76)
Cognitive ability score at draft (Borge Prien)^d^	44.04 (8.01)	40.14 (6.30)
Depression at SCID (%)	2.31	54.55

SCID = Structured Clinical Interview for DSM-IV-TR Axis-I Disorder; PCL-C = Post-Traumatic Stress Disorder Checklist Civilian; DIS = Danger/Injury Scale; CES = Combat Exposure Scale; PANAS = Positive And Negative Affect Schedule; PJ/IJ = Procedural and Interactional Justice.

^a^Average and standard deviation (*SD*) of subgroups, unless percentage is indicated (%).

^b^Whilst gender is included as predeployment, data on gender came from 2½–3 years postdeployment.

^c^The data missing vary between measures.

^d^The Borge Prien test is a validated cognitive ability test used in Danish drafts.

### 3.1. Primary analysis: PJ/IJ, SCID

For the primary analysis, introducing the variables on deployment related traumatic exposure and PJ/IJ into the ‘basic model’ (Table 3) showed that a more intense environment of possible traumatic events, as measured with the DIS (odds ratio [OR] = 1.16, 95% CI 1.01–1.35) was a significant predictor of PTSD on the SCID. This was also the case for better perceptions of PJ/IJ in relation to immediate superiors during deployment (OR = 0.85, 95% CI 0.74–0.96). Combat-related exposure measured with the CES or having additional deployments in the time between homecoming from this mission and the SCID were, however, not related.

**Table 3.** Multivariate relation with PTSD at the SCID in logistic regression models, PJ/IJ during deployment.

PJ/IJ and PANAS measurement	During deployment		
Model description	Basic model	Main model	Predeployment depressive model
Predeployment variables			
Age		1.04 (0.95–1.13)	1.05 (0.96–1.22)
Female^a^		1.04 (0.13–5.24)	0.63 (0.03–4.61)
PCL-C score		1.01 (0.95–1.08)	0.93 (0.82–1.04)
Depressive symptoms score			1.07 (0.94–1.22)
During deployment variables			
DIS	1.16 (1.01–1.35)*	1.08 (0.92–1.29)	1.00 (0.83–1.19)
CES	1.01 (0.91–1.12)	1.00 (0.89–1.12)	1.05 (0.93–1.20)
PANAS positive score		1.05 (0.99–1.13)	1.07 (1.00–1.17)
PANAS negative score		1.08 (0.97–1.19)	1.09 (0.98–1.21)
PJ/IJ	0.85 (0.74–0.96)*	0.86 (0.75–0.98)*	0.85 (0.73–0.98)*
Postdeployment variables			
Additional deployment before SCID	0.71 (0.28–1.83)	0.69 (0.25–1.94)	0.76 (0.26–2.30)
Additional trauma exposure before SCID		1.37 (1.05–1.80)*	1.44 (1.08–1.94)*
Nagelkerke (pseudo) R^2^	0.14	0.24	0.26
−2 Log likelihood	131.72	119.13	108.75
χ^2^ (df)	15.91 (4)**	28.50 (10)**	28.40 (11)*

OR = Odds ratio; SCID = Structured Clinical Interview for DSM-IV-TR Axis-I Disorder; PCL-C = Post-Traumatic Stress Disorder Checklist Civilian; DIS = Danger/Injury Scale; CES = Combat Exposure Scale; PANAS = Positive And Negative Affect Schedule; PJ/IJ = Procedural and Interactional Justice.

*p < .05, ** *p* < .01.

^a^Whilst gender is included as predeployment, data on gender came from 2½–3 years postdeployment.

The ‘main model’ further included the risk/resilience variables of age, gender, predeployment PCL-C score and positive and negative state affectivity. However, of these only the number of additional traumatic event (OR = 1.37, 95% CI 1.05–1.80) and perceptions of PJ/IJ (OR = 0.86, 95% CI 0.75–0.98) maintained a significant relationship with PTSD on the SCID. Including the predeployment depressive symptoms into the ‘Predeployment depressive model’ did not overall change the significant relationships with number of traumatic events (OR = 1.44, 95% CI 1.08–1.94) or PJ/IJ (OR = 0.85, 95% CI 0.73–0.98). Positive affectivity during deployment, measured with the PANAS, however, bordered on having a significant relation to a higher risk of PTSD on the SCID in this model (OR = 1.07, 95% CI 1.00–1.17).

The supplementary primary analysis of the relation of PJ/IJ and PANAS with PTSD on the SCID, throughout the deployment cycle, showed that a better perception of PJ/IJ 1–2 months before deployment (OR = 0.83, 95% CI 0.71–0.95) was also a significant predictor for lower odds of PTSD. This was, however, not the case for the measure 1–3 weeks after deployment (OR = 0.97, 95% CI 0.85–1.11) (results not shown).

### 3.2. Secondary analyses: PJ/IJ, screening levels of PCL-C

The secondary analysis introduced predictors of the main model into four ‘screening models’, to test the relation with having screening levels of PTSD symptoms measured with the PCL-C at four measures postdeployment (Supplemental data Table S3). Better perceptions of PJ/IJ bordered on having a significant relation to PTSD symptom levels at 1–3 weeks and 2–3 months after deployment, but not at 7–8 months or 2½–3 years. A more negative affectivity during deployment showed a significant relation to screening levels of PTSD on the PCL-C at 1–3 weeks and 7–8 months, but not at 2–3 months or 2½–3 years. Finally, a consistently significant relationship of higher predeployment PTSD symptoms with PTSD at screening levels was found, in the range of OR 1.08–1.12, but the outcome at 7–8 months only bordered on significance. None of the models showed significant relationships to any of the other risk/resilience factors. Differences in the zero-order correlation and the regression model prediction by the predeployment PCL-C measure of the SCID PTSD and the screening levels of PTSD symptoms, even when obtained at the same time point, could indicate overcontrol.

When excluding predeployment PTSD symptoms, measured with the same scale, the PJ/IJ measure was found to predict PTSD at screening levels, at 1–3 weeks (OR = 0.87, 95% CI 0.76–0.98) and 2–3 months (OR = 0.83, 95% CI 0.69–0.99) postdeployment (Supplemental data Table S4).

## 4. Discussion

The present multiwave cohort study examined the prospective relationship between PJ/IJ in relation to immediate superiors, as perceived by soldiers deployed into a combat zone in Helmand Province in Afghanistan in 2009, and PTSD. The PTSD outcomes were diagnosis with the SCID approximately 2½ years after homecoming and reporting a screening level of self-reported PTSD symptoms at four different time points after homecoming. Whilst studies of OJ or soldiers’ perceptions of leadership in relation to PTSD in military settings are not new, this is, to our knowledge, the first study to examine the prospective relationships between soldiers’ perception of aspects of OJ during deployment to a war zone and postdeployment PTSD.

### 4.1. Main findings, PJ/IJ

In line with our hypothesis, the result showed that experiencing higher levels of PJ/IJ in relation to an immediate superior during military deployment is related to lower odds of meeting the criteria for PTSD on the SCID 2½–3 years after homecoming. This relation was found while accounting for gender and age, predeployment PTSD and depressive symptom scores, perceived levels of general traumatic exposure and combat exposure during deployment, and positive and negative state affectivity while judging PJ/IJ. Further analysis suggested that such a relation also existed when PJ/IJ was measured before deployment. Measures of PJ/IJ were overall stable across the deployment cycle, even though some variation must be expected given the major changes that occur at home compared to that in the war zone (data not shown). However, we did not find the perceptions of PJ/IJ immediately after deployment to relate to PTSD on the SCID. While this could pertain to the measurement point at 1–3 week after homecoming, these results may also be explained by the theory of conservation of resources. This suggests that positive and negative aspects of leadership spiral over time into more or fewer resources and result in resilience or vulnerability (Hobfoll, 2001; McGurk et al., 2014). We do find that the PJ/IJ measurements before and during deployment, which allows for the most risk time, are the most closely related to a risk of PTSD on the SCID.

Further, the secondary analyses of the relation between PJ/IJ during deployment and screening levels of PTSD symptoms at the four postdeployment time points showed some of the same tendencies. However, a significant relation to PJ/IJ only occurred when we excluded the predeployment PCL-C as a predictor, given the possibility of overcontrol. We can therefore not make a firm conclusion about the relation to screening levels of self-reported PTSD symptoms.

Overall, the results of the relationship between PJ/IJ and PTSD do suggest that experience of lower or higher levels of PJ/IJ in relation to an immediate superior, throughout deployment into an active combat zone, may be a genuine risk/resilience factor for postdeployment PTSD diagnoses, for soldiers without their own leadership obligations.

The ‘alarm-system perspective’ from the OJ literature may provide a valid theoretical explanation for the relationship found, since perception of PJ/IJ may act as a substitute for real information on dangers, which may thus calm down, or give rise to, further negative reactions for the deployed soldier. An alternative explanation for the relationship may, however, relate to the individual’s relations with the group. Group cohesion and support have been extensively studied in relation to the risk of postdeployment mental health problems (Du Preez et al., 2012; Ramchand et al., 2015; Xue et al., 2015). In the justice literature, PJ has been suggested to be carrying information about the individual’s hierarchical position or belongingness to the group (Blader & Tyler, 2015). An alternative explanation for our results could, therefore, be that the perceptions of PJ/IJ measured in our study also serve as a proxy for an actual supportive effect of leadership on group cohesion and support (Ramchand et al., 2015). Finally, leadership behaviours have previously been found to influence stigma and barriers to care (Britt et al., 2012); perceptions of PJ/IJ may thus be linked to PTSD through help-seeking behaviours. Future research should consider including tests of such mediations with valid measures.

### 4.2. Other findings

In line with the literature, our study found trauma exposure in the time after deployment to be strongly prospectively related to PTSD. Further, traumatic exposure during deployment was related to the odds of having PTSD on the SCID 2½ years later. However, the latter was only significant when nontraumatic measures and postdeployment trauma were excluded. The measure of direct combat exposure, the CES, was not related to PTSD on the SCID or with screening levels of PTSD symptoms at any time. While these negative results are unexpected, it is in line with previous results, showing no relation to PTSD symptom fluctuations or reintegration problems in the population (Andersen et al., 2014; Karstoft, Armour, Andersen, Bertelsen, & Madsen, 2015). There was further a borderline relationship between a more positive state affectivity during deployment and higher odds of the presence of PTSD on the SCID, and a significant relation between negative affectivity and PTSD at screening levels.

### 4.3. Implications

This study extends prior research (Castro & McGurk, 2007; Du Preez et al., 2012; Iversen et al., 2008; Jones et al., 2012; McGurk et al., 2014), that linked perceptions of aspects of leadership with a risk of PTSD, by suggesting that perceptions of leadership may be understood within the OJ framework. These findings may have theoretical implications for how we may understand and measure perceptions of leadership.

Furthermore, the findings extend research that has related OJ, both theoretically and empirically, to mental health outcomes by showing that possible protective effects against mental health problems may include protection against problems resulting from traumatic events (Ford & Huang, 2014; Ndjaboué et al., 2012; Robbins et al., 2012).

Pertaining to interventions, it has been suggested that there is a lack of research on interventions aimed at strengthening OJ (Greenberg, 2009). Soldier populations deployed into war zones may, however, provide perfect settings for future studies for several reasons: (1) superiors and subordinates prepare for deployments, which are (2) of limited length in (3) an environment controlled by the organization. Finally, such interventions may be of interest, as they possibly prevent the development of a serious mental health outcome, which often renders soldiers unfit for further deployments.

The clinical implications may be twofold. First, in contrast to other static risk and resilience factors such as gender, mental problems and previous or new traumatic exposure (Xue et al., 2015), the perceptions of PJ/IJ in relation to immediate superiors may be an actual modifiable factor, susceptible to change both before and during deployment. Also, understanding perceptions of leadership during military deployment through an OJ perspective allows for interventions and predeployment programmes to draw on the existing results of this literature, however limited they are. Second, given the findings of a relationship already with predeployment perceptions of PJ/IJ, such predeployment measures may raise some awareness as to who and which groups may be at an elevated risk. Finally, the findings of a strong relationship between the experience of possibly traumatic events after military deployment and PTSD should encourage future research to also account for exposure in the time after homecoming, before PTSD is identified.

### 4.4. Strengths and limitations

A strength of the present study was the multiwave longitudinal design throughout deployment to an active war zone, allowing us to draw inferences based on pre-, peri- and postdeployment data, and allowing thorough testing for possible confounding effects. Furthermore, the use of a PTSD outcome based on the SCID allowed us to use independent judgements about the presence of possible PTSD, in contrast to self-assessed symptom levels only. Finally, the 6-item scale of PJ/IJ was validated using the somewhat strict requirements of the Rasch validation.

Several limitations of the study must also be acknowledged. First, the small sample size (*n = *243) and the few cases of PTSD based on the SCID (*n = *22) should be acknowledged. This restricted the possibilities of inferring results from possible predictors beyond the traumatic exposure–OJ–PTSD relationship, such as interactions between measures like PJ/IJ and trauma exposures. Second, the current study is based on a secondary analysis of data obtained for general research purposes, for which reason the questions used for the PJ/IJ measure were not originally included for this purpose and why the scale validation using the Rasch procedures was conducted in this population only. Furthermore, it was not possible to investigate the aspect of distributive justice. Thus, while the PJ/IJ measure did show fine properties, future studies on OJ in a deployed military environment should therefore also consider using other tailored questionnaires, such as that of Olsen et al. (2012). Third, although we do not suspect it affected our findings, the age differences of those participating in the SCID should be noted. Fourth, our choice of using the DIS and CES measures during deployment, due to possible bias in the originally intended data, excluded events occurring in the second part of the deployment. Also, a self-reported measure of ‘further deployments’ was included and, although this could control for additional traumatic exposure, the number or duration was not available and the non-impact of this measure may, in fact, cover two opposite effects: (1) persons taking on additional tours may have lower symptom levels of PTSD, and (2) additional deployments may result in a higher risk of getting PTSD. Finally, the inclusion procedure required that only soldiers without leadership obligations were included, and we do therefore not know if similar relations are found also for soldiers with leadership responsibilities. Given their position, these higher-ranked soldiers may be more well informed and have more of a say in how to tackle traumatizing situations, and their perceptions of justice may, therefore, add the kind of protection suggested in the ‘alarm-system perspective’.

## 5. Conclusions

In summary, this study examined the longitudinal relationship between perceptions of PJ/IJ in relation to immediate superiors and the risk of developing PTSD after deployment amongst Danish soldiers without their own leadership obligations, operating in Helmand Province in Afghanistan in 2009. As hypothesized, we found an association between higher levels of PJ/IJ during deployment and lower odds of developing PTSD 2½–3 years after deployment. This association was found after controlling for factors such as predeployment PTSD symptoms, positive and negative state affectivity, experiences of traumatic events and combat exposure during deployment, and traumatic events after homecoming. These findings suggest that experiencing higher levels of PJ/IJ in relation to immediate superiors during deployment may independently protect soldiers deployed into an active war zone against developing postdeployment PTSD. We further suggest that soldiers’ relationships with immediate superiors during deployment may be understood within the OJ framework. Modifying factors that lead to better perceptions of PJ/IJ in relation to an immediate superior may thus possibly aid in the prevention of PTSD cases. However, given the limitations of the study, these conclusions must be considered provisional.
