# Supplementary material for: Leadership and post-traumatic stress disorder: are soldiers’ perceptions of organizational justice during deployment protective?
Source: Eur J Psychotraumatol. 2018 Apr 4;9(1):1449558. doi: 10.1080/20008198.2018.1449558 (PMC5912440; doi:10.1080/20008198.2018.1449558)
Supplement: Supplementary material [file ZEPT_A_1449558_SM4236.docx]

# Supplementary material

## Procedural and Interactional Justice scale validation

The procedural and interactional justice (PJ/IJ) scale was validated using graphical log-linear Rasch modelling (GLLRM). For an overview of the general Rasch modelling, see for example Karstoft, Nielsen & Nielsen (2017), where such procedures previously have been used to validate a depression scale in the present sample. The aim of the Rasch modelling is to assure that the measure functions psychometrically adequately in the sample (e.g. that the scale measures one thing only and does so independently of scoring patters). The procedures form an internal validation, as it is not validated on dependence with external factors. Rasch procedures can therefore be considered an advantage in a situation, like the present, where the data are unique, but it is still necessary that the scales psychometric properties are appropriate.

The validation procedure was conducted using GLLRM through Digram (v. 3.36.0) (Kreiner, 2003) on soldiers (N=359) from the current sample, who were without leadership obligations and who answered the questions on PJ/IJ during deployment. Items came from an 11-item Danish validated scale covering judgements of fairness and quality of leadership employed by an immediate superior, which was originally developed and has been used as a part of a larger survey instrument by the Danish military (Borup-Nielsen, Kousgaard, & Rieneck, 1973). From this scale, eight initial items were selected, based on the questions used to measure the PJ and the subcomponents of IJ (i.e. Interpersonal and Informational Justice) in a population of Norwegian military officers (Olsen, Myrseth, Eidhamar, & Hystad, 2012). The scales were tested for fit in the GLLRM, including differential item functioning in relation to gender, being in an infantry unit and having had previous deployments. Through these processes, two of the eight selected items were discarded due to evidence of high local dependence on other items, in combination with the wording of the two items, which related to the specific tasks the soldiers performed, which would vary across the soldiers’ functioning. An acceptable model still had some local dependence, and, given evidence of differential item functioning, a scoring equation was induced so scores for non-infantry units overall were adjusted slightly compared to those for infantry units. But, the score equations were only minor and did not affect the average scores between the groups.

The resulting 6-item scale contained the following questions in Danish (first author’s translation): ‘Do you find that your immediate superior has an interest in what goes on amongst the employees?’, ‘Do you find that your immediate superior to a reasonable degree takes the individual into account?’, ‘Do you find that your immediate superior is treating you in a fair manner?’, ‘Do you find that your immediate superior contributes to keeping an open discussion about work-related topics?’, ‘Does your immediate superior show you that he/she values the work you are doing?’ and ‘Do you think that your immediate superior is willing to pass on your wishes and views?’. Based the wording of two answer categories, which distinguished only between ‘No, only slightly’ and ‘To a minor degree’ in combination with results from a procedure in Digram testing scoring patterns, the two answer categories were collapsed. The scale was then scored ‘No, only slightly’/‘To a minor degree’ (0), ‘Yes, to some degree’ (1) and ‘Yes, to a high degree’ (2).

For the model the standard error of the mean for the scale theta was in the range of 0.7-0.8, whilst Cronbach’s α was in the range of 0.88-0.99 between the subgroups tested. Based on this, care should be taken before using it for individual judgements of procedural and interactional justice levels, e.g. such as at the selection of soldiers. Nonetheless, the scale can be considered excellent for research purposes, such as the present case.

# Literature

Borup-Nielsen, S., Kousgaard, E., & Rieneck, B. (1973). *Measurement of attitudes in the Defence (Original title, Danish: Holdningsmålinger i forsvaret)*. Copenhagen: Danish Armed Forces Psychological Service.

Karstoft, K.-I., Nielsen, A. B. S., & Nielsen, T. (2017). Assessment of depression in veterans across missions: a validity study using Rasch measurement models. *European Journal of Psychotraumatology*, *8*(1), 1326798. doi:10.1080/20008198.2017.1326798

Kreiner, S. (2003). *Introduction to DIGRAM. Research report 03/10*. Copenhagen: Dept. of Biostatistics, University of Copenhagen. Retrieved from http://publichealth.ku.dk/sections/biostatistics/reports/2003/rr-03-10.doc

Olsen, O. K., Myrseth, H., Eidhamar, A., & Hystad, S. W. (2012). Psychometric Properties of a Four-Component Norwegian Organizational Justice Scale. *Psychological Reports*, *110*(2), 571–588. doi:10.2466/01.08.14.PR0.110.2.571-588

## Timing of study variables

## Supplementary Table S1: Timing of study variables, the use and population size at each time

|  | **Before deployment** | **During deployment** | **After deployment** | | | |
| --- | --- | --- | --- | --- | --- | --- |
|  | 1-2 months before | Approx. 3 months into | 1-3 weeks | 2-3 months | 7-8 months | 2½-3 years |
| **Population size** | n=243 | n=243 | n=209 | n=144 | n=122 | n=243 |
| **Predictor variables** |  |  |  |  |  |  |
| Age | 1, 2, 3, 4 |  |  |  |  |  |
| Female^b^ |  |  |  |  |  | 1, 2, 3, 4 |
| PCL-C score | 1, 2, 3, 4 |  |  |  |  |  |
| Depressive symptoms score | 1 |  |  |  |  |  |
| DIS |  | 1, 2, 3, 4 |  |  |  |  |
| CES |  | 1, 2, 3, 4 |  |  |  |  |
| PANAS negative score | 2 | 1 | 3 |  |  |  |
| PANAS positive score | 2 | 1 | 3 |  |  |  |
| PJ/IJ | 2 | 1 | 3 |  |  |  |
| Additional deployment before SCID |  |  |  |  |  | 1, 2, 3 |
| Additional trauma exposure before SCID |  |  |  |  |  | 1, 2, 3 |
| **Outcome variables** |  |  |  |  |  |  |
| SCID, No PTSD vs. PTSD |  |  |  |  |  | 1, 2, 3 |
| PCL-C, Low vs. Screening level |  |  | 4 | 4 | 4 | 4 |
| SCID = Structured Clinical Interview for DSM-IV-TR Axis-I Disorder; PCL-C = Post-traumatic Stress Disorder Checklist, Civilian; DIS = Danger/Injury Scale; CES = Combat Exposure Scale; PANAS = Positive and Negative Affect Schedule; PJ/IJ = Procedural and Interactional Justice | | | | | | |
| 1: Variable included in the main model analysis | | |  |  |  |  |
| 2: Variable included in the supplementary primary analysis, PJ/IJ and PANAS measured before deployment | | | |  |  |  |
| 3: Variable included in the supplementary primary analysis, PJ/IJ and PANAS measured after deploynent | | |  |  |  |  |
| 4: Variable included in the secondary analyses with subclinical PCL-level as outcome | | |  |  |  |  |

## Zero-order Spearman correlations between variables for the main model

| *Supplementary Table, S2: Zero-order Spearman correlations between variables for the population in the primary model (n=243)* | | | | | | | | | | | | | | | | |  |  |  |  |  |  |  |  |  |  |
| --- | --- | --- | --- | --- | --- | --- | --- | --- | --- | --- | --- | --- | --- | --- | --- | --- | --- | --- | --- | --- | --- | --- | --- | --- | --- | --- |
|  |  |  |  |  |  |  |  |  |  |  |  |  |  |  |  |  |  |  |  |  |  |  |  |  |  |  |
|  | Outcomes | SCID PTSD diagnosis | PCL-C Screening, 1-3 weeks after | PCL-C Screening, 2-3 months after | PCL-C Screening, 7-8 months after | PCL-C Screening level, 2½-3 years after | Predictors, Main | Age | Female | PCL-C score | Depressive symptoms score | DIS | CES | PANAS positive score, during | PANAS negative score, during | PJ/IJ, during | Additional deployment before SCID | Additional trauma exposure before SCID | Predictors, PJ/IJ before | PANAS positive score, before | PANAS negative score, before | PJ/IJ, before | Predictors, PJ/IJ after | PANAS positive score, after | PANAS negative score, after | PJ/IJ, after |
| Outcomes |  |  |  |  |  |  |  |  |  |  |  |  |  |  |  |  |  |  |  |  |  |  |  |  |  |  |
| SCID PTSD diagnosis |  |  | ***0.22***** | ***0.23***** | ***0.44***** | ***0.44***** |  | 0.04 | 0.01 | 0.13 | 0.02 | ***0.18***** | 0.11 | ***0.16**** | ***0.14**** | ***-0.15**** | -0.05 | ***0.25***** |  | 0.01 | 0.04 | ***-0.15**** |  | 0.07 | 0.13 | -0.01 |
| PCL-C Screening, 1-3 weeks after |  | ***0.22***** | | ***0.54***** | ***0.34***** | ***0.25***** |  | 0.01 | 0.12 | ***0.36***** | ***0.33***** | ***0.17**** | 0.11 | 0.02 | ***0.37***** | ***-0.14**** | 0.03 | 0.05 |  | -0.04 | ***0.28***** | ***-0.19***** |  | 0.05 | ***0.45***** | -0.13 |
| PCL-C Screening, 2-3 months after |  | ***0.23***** | ***0.54***** | | ***0.50***** | ***0.25***** |  | 0.08 | 0.07 | ***0.33***** | 0.15 | 0.13 | -0.01 | -0.03 | ***0.20**** | -0.15 | -0.11 | -0.01 |  | -0.03 | ***0.20**** | -0.16 |  | -0.05 | ***0.27***** | -0.17 |
| PCL-C Screening, 7-8 months after |  | ***0.44***** | ***0.34***** | ***0.50***** | | ***0.33***** |  | -0.06 | ***0.19**** | ***0.40***** | ***0.29***** | ***0.27***** | ***0.19**** | 0.03 | ***0.39***** | -0.10 | -0.15 | 0.11 |  | -0.06 | 0.14 | ***-0.20**** |  | -0.07 | ***0.29***** | 0.03 |
| PCL-C Screening, 2½-3 years after |  | ***0.44***** | ***0.25***** | ***0.25***** | ***0.33***** | |  | -0.04 | 0.02 | ***0.35***** | ***0.17**** | ***0.16**** | ***0.14**** | 0.09 | ***0.20***** | 0.00 | 0.01 | ***0.28***** |  | -0.01 | 0.07 | -0.09 |  | 0.02 | ***0.21***** | 0.00 |
| Predictors, Main Model |  |  |  |  |  |  |  |  |  |  |  |  |  |  |  |  |  |  |  |  |  |  |  |  |  |  |
| Age |  | 0.04 | 0.01 | 0.08 | -0.06 | -0.04 |  |  | -0.09 | ***-0.21***** | ***-0.21***** | ***-0.14**** | ***-0.21***** | 0.02 | ***-0.26***** | 0.03 | 0.05 | ***-0.14**** |  | 0.01 | ***-0.18***** | 0.12 |  | 0.04 | -0.08 | 0.04 |
| Female |  | 0.01 | 0.12 | 0.07 | ***0.19**** | 0.02 |  | -0.09 |  | 0.11 | 0.10 | -0.07 | ***-0.18***** | 0.02 | 0.10 | -0.05 | -0.02 | 0.06 |  | -0.06 | ***0.15**** | -0.02 |  | -0.02 | 0.11 | 0.02 |
| PCL-C score |  | 0.13 | ***0.36***** | ***0.33***** | ***0.40***** | ***0.35***** |  | ***-0.21***** | 0.11 |  | ***0.69***** | ***0.23***** | ***0.19***** | -0.06 | ***0.40***** | -0.12 | -0.02 | ***0.21***** |  | ***-0.14**** | ***0.55***** | ***-0.25***** |  | -0.05 | ***0.43***** | -0.03 |
| Depressive symptoms score |  | 0.02 | ***0.33***** | 0.15 | ***0.29***** | ***0.17**** |  | ***-0.21***** | 0.10 | ***0.69***** | | ***0.19***** | 0.12 | ***-0.13**** | ***0.41***** | -0.07 | 0.04 | 0.12 |  | ***-0.22***** | ***0.57***** | ***-0.25***** |  | -0.14 | ***0.32***** | -0.01 |
| DIS |  | ***0.18***** | ***0.17**** | 0.13 | ***0.27***** | ***0.16**** |  | ***-0.14**** | -0.07 | ***0.23***** | ***0.19***** | | ***0.74***** | ***0.17***** | ***0.26***** | 0.05 | -0.03 | ***0.26***** |  | ***0.22***** | ***0.26***** | -0.03 |  | 0.11 | ***0.33***** | 0.06 |
| CES |  | 0.11 | 0.11 | -0.01 | ***0.19**** | ***0.14**** |  | ***-0.21***** | ***-0.18***** | ***0.19***** | 0.12 | ***0.74***** | | ***0.15**** | ***0.23***** | -0.01 | -0.04 | ***0.18***** |  | ***0.32***** | ***0.18***** | 0.03 |  | ***0.19***** | ***0.26***** | 0.05 |
| PANAS positive score, during |  | ***0.16**** | 0.02 | -0.03 | 0.03 | 0.09 |  | 0.02 | 0.02 | -0.06 | ***-0.13**** | ***0.17***** | ***0.15**** |  | 0.03 | 0.11 | ***-0.14**** | ***0.17***** |  | ***0.52***** | -0.08 | 0.09 |  | ***0.56***** | 0.12 | ***0.14**** |
| PANAS negative score, during |  | ***0.14**** | ***0.37***** | ***0.20**** | ***0.39***** | ***0.20***** |  | ***-0.26***** | 0.10 | ***0.40***** | ***0.41***** | ***0.26***** | ***0.23***** | 0.03 |  | -0.08 | 0.06 | 0.11 |  | 0.01 | ***0.46***** | -0.12 |  | 0.01 | ***0.53***** | -0.06 |
| PJ/IJ, during |  | ***-0.15**** | ***-0.14**** | -0.15 | -0.10 | 0.00 |  | 0.03 | -0.05 | -0.12 | -0.07 | 0.05 | -0.01 | 0.11 | -0.08 |  | -0.06 | -0.01 |  | 0.07 | 0.04 | ***0.45***** |  | 0.04 | 0.02 | ***0.59***** |
| Addtional deployment before SCID |  | -0.05 | 0.03 | -0.11 | -0.15 | 0.01 |  | 0.05 | -0.02 | -0.02 | 0.04 | -0.03 | -0.04 | ***-0.14**** | 0.06 | -0.06 |  | -0.05 |  | ***-0.13**** | -0.07 | -0.09 |  | -0.04 | 0.00 | -0.02 |
| Additional trauma exposure before SCID |  | ***0.25***** | 0.05 | -0.01 | 0.11 | ***0.28***** |  | ***-0.14**** | 0.06 | ***0.21***** | 0.12 | ***0.26***** | ***0.18***** | ***0.17***** | 0.11 | -0.01 | -0.05 |  |  | 0.12 | ***0.14**** | -0.02 |  | 0.03 | 0.08 | 0.04 |
| Predictors, PJ/IJ before |  |  |  |  |  |  |  |  |  |  |  |  |  |  |  |  |  |  |  |  |  |  |  |  |  |  |
| PANAS positive score, before |  | 0.01 | -0.04 | -0.03 | -0.06 | -0.01 |  | 0.01 | -0.06 | ***-0.14**** | ***-0.22***** | ***0.22***** | ***0.32***** | ***0.52***** | ***0.01***** | 0.07 | ***-0.13**** | ***0.12*** |  |  | -0.01 | ***0.15**** |  | ***0.46***** | 0.07 | 0.09 |
| PANAS negative score, before |  | 0.04 | ***0.28***** | ***0.20**** | 0.14 | 0.07 |  | ***-0.18***** | ***0.15**** | ***0.55***** | ***0.57***** | ***0.26***** | ***0.18***** | -0.08 | 0.46 | 0.04 | -0.07 | ***0.14**** |  | -0.01 |  | -0.08 |  | 0.03 | ***0.57***** | 0.11 |
| PJ/IJ, before |  | ***-0.15**** | ***-0.19***** | -0.16 | ***-0.20**** | -0.09 |  | 0.12 | -0.02 | ***-0.25***** | ***-0.25***** | -0.03 | 0.03 | 0.09 | -0.12 | ***0.45***** | -0.09 | -0.02 |  | ***0.15**** | -0.08 |  |  | 0.10 | ***-0.15**** | ***0.34***** |
| Predictors, PJ/IJ after |  |  |  |  |  |  |  |  |  |  |  |  |  |  |  |  |  |  |  |  |  |  |  |  |  |  |
| PANAS positive score, after |  | 0.07 | 0.05 | -0.05 | -0.07 | 0.02 |  | 0.04 | -0.02 | -0.05 | -0.14 | 0.11 | ***0.19***** | 0.56 | 0.01 | 0.04 | -0.04 | 0.03 |  | ***0.46***** | 0.03 | 0.10 |  |  | ***0.16**** | 0.11 |
| PANAS negative score, after |  | 0.13 | ***0.45***** | ***0.27***** | ***0.29***** | ***0.21***** |  | -0.08 | 0.11 | ***0.43***** | ***0.32***** | ***0.33***** | ***0.26***** | ***0.12***** | ***0.53***** | 0.02 | 0.00 | 0.08 |  | 0.07 | ***0.57***** | ***-0.15**** |  | ***0.16**** |  | 0.01 |
| PJ/IJ, before |  | -0.01 | -0.13 | -0.17 | 0.03 | 0.00 |  | 0.04 | 0.02 | -0.03 | -0.01 | 0.06 | 0.05 | 0.14* | -0.06 | ***0.59***** | -0.02 | 0.04 |  | 0.09 | 0.11 | ***0.34***** |  | 0.11 | 0.01 |  |
| SCID = Structured Clinical Interview for DSM-IV-TR Axis-I Disorder; PCL-C = Post-traumatic Stress Disorder Checklist, Civilian; DIS = Danger/Injury Scale; CES = Combat Exposure Scale; PANAS = Positive and Negative Affect Schedule; PJ/IJ = Procedural and Interactional Justice | | | | | | | | | | | |  |  |  |  |  |  |  |  |  |  |  |  |  |  |  |
| *P < 0.05, **P < 0.01 | | | | | | | | | | | |  |  |  |  |  |  |  |  |  |  |  |  |  |  |  |

## Supplementary tables: PJ/IJ during, PCL-C outcome at screening levels four times postdeployment.

| *Supplementary Table, S3: Multivariate relation with moderate to high PTSD symptoms in logistic regression models, PJ/IJ during deployment* | | | | | | |  | |
| --- | --- | --- | --- | --- | --- | --- | --- | --- |
|  |  | | | | | | | |
| **PJ/IJ and PANAS, measurement** | *During deployment* | | | | | | | |
| OUTCOME^a^ | Low symptom (n=178) vs. Screening level (n=31), | Low symptom (n=132) vs. Screening level (n=12), | | Low symptom (n=100) vs. Screening level (n=22), | | Low symptom (n=174) vs. Screening level (n=69), | |  |
|  | 1-3 weeks after | 2-3 months after | | 7-8 months after | | 2½-3 years after | |  |
| Predeployment measures |  |  | |  | |  | |  |
| Age | 1.08 (0.99 - 1.17) | 1.08 (0.96 - 1.20) | | 1.05 (0.95 - 1.15) | | 0.99 (0.94 - 1.04) | |  |
| Female^b^ | 3.09 (0.68 - 12.37) | 0.58 (0.04 - 4.89) | | 6.79 (0.61 - 73.17) | | 0.83 (0.23 - 2.63) | |  |
| PCL-C score | 1.08 (1.02 - 1.14)** | 1.12 (1.04 - 1.22)** | | 1.08 (1.00 - 1.17) | | 1.08 (1.04 - 1.13)** | |  |
| During deployment measures |  |  | |  | |  | |  |
| DIS | 1.04 (0.89 - 1.21) | 1.16 (0.92 - 1.51) | | 1.15 (0.97 - 1.38) | | 1.03 (0.93 - 1.13) | |  |
| CES | 1.02 (0.92 - 1.14) | 0.90 (0.74 - 1.05) | | 0.99 (0.86 - 1.14) | | 1.02 (0.95 - 1.09) | |  |
| PANAS positive score | 1.00 (0.94 - 1.06) | 1.02 (0.92 - 1.14) | | 1.00 (0.93 - 1.09) | | 1.02 (0.98 - 1.07) | |  |
| PANAS negative score | 1.19 (1.09 - 1.31)** | 1.01 (0.84 - 1.18) | | 1.16 (1.05 - 1.31)** | | 1.02 (0.95 - 1.09) | |  |
| PJ/IJ | 0.88 (0.77 - 1.00) | 0.84 (0.69 - 1.01) | | 0.94 (0.79 - 1.10) | | 1.01 (0.93 - 1.10) | |  |
|  |  |  | |  | |  | |  |
| Nagelkerke (pseudo) R^2^ | 0.36 | 0.32 | | 0.35 | | 0.15 | |  |
| -2 Log likelihood | 127.85 | 60.58 | | 86.17 | | 262.22 | |  |
| χ^2^ (df) | 47.63 (8)** | 22.03 (8)** | | 28.97 (8)** | | 27.75 (8)** | |  |
| OR = Odds ratio; SCID = Structured Clinical Interview for DSM-IV-TR Axis-I Disorder; PCL-C = Post-traumatic Stress Disorder Checklist, Civilian; DIS = Danger/Injury Scale; CES = Combat Exposure Scale; PANAS = Positive and Negative Affect Schedule; PJ/IJ = Procedural and Interactional Justice | | | | | | | | |
| *P < 0.05, **P < 0.01 |  | |  | |  | |  | |
| ^a^Differences in population size are due to missing data at measurement. | | |  | |  | |  | |
| ^b^Whilst gender is included as predeployment, data on gender came from 2½-3y postdeployment. | | | | |  | |  | |

| *Supplementary Table, S4: Multivariate relation with moderate to high PTSD symptoms in logistic regression models, PJ/IJ during deployment, PCL-C 'before' not included* | | | | |
| --- | --- | --- | --- | --- |
| **PJ/IJ and PANAS, measurement** | *During deployment* | | | |
| OUTCOME^a^ | Low symptom (n=178) vs. Screening level (n=31), | Low symptom (n=132) vs. Screening level (n=12), | Low symptom (n=100) vs. Screening level (n=22), | Low symptom (n=174) vs. Screening level (n=69), |
|  | 1-3 weeks after | 2-3 months after | 7-8 months after | 2½-3 years after |
| Pre-deployment measures |  |  |  |  |
| Age | 1.06 (0.97 - 1.15) | 1.07 (0.95 - 1.18) | 1.04 (0.95 - 1.14) | 0.98 (0.93 - 1.04) |
| Female^b^ | 4.19 (1.07 - 15.28)* | 1.53 (0.18 - 9.29) | 9.07 (1.12 - 81.58)* | 1.21 (0.39 - 3.45) |
| During deployment measures |  |  |  |  |
| DIS | 1.1 (0.95 - 1.27) | 1.25 (1.01 - 1.57)* | 1.14 (0.97 - 1.36) | 1.06 (0.96 - 1.16) |
| CES | 1.00 (0.90 - 1.10) | 0.88 (0.74 - 1.02) | 1.02 (0.89 - 1.16) | 1.01 (0.94 - 1.08) |
| PANAS positive score | 0.99 (0.94 - 1.05) | 0.99 (0.90 - 1.08) | 1.00 (0.93 - 1.08) | 1.02 (0.98 - 1.06) |
| PANAS negative score | 1.23 (1.13 - 1.36)** | 1.11 (0.97 - 1.25) | 1.20 (1.08 - 1.34)** | 1.07 (1.00 - 1.13)* |
| PJ/IJ | 0.87 (0.76 - 0.98)* | 0.83 (0.69 - 0.99)* | 0.94 (0.80 - 1.10) | 0.99 (0.92 - 1.08) |
|  |  |  |  |  |
| Nagelkerke (pseudo) R^2^ | 0.31 | 0.21 | 0.31 | 0.08 |
| -2 Log likelihood | 135.67 | 68.988 | 89.794 | 276.01 |
| χ^2^ (df) | 39.81 (7)** | 13.62 (7) | 25.35 (7)** | 13.96 (7) |
| OR = Odds ratio; SCID = Structured Clinical Interview for DSM-IV-TR Axis-I Disorder; PCL-C = Post-traumatic Stress Disorder Checklist, Civilian; DIS = Danger/Injury Scale; CES = Combat Exposure Scale; PANAS = Positive and Negative Affect Schedule; PJ/IJ = Procedural and Interactional Justice | | | | |
| *P < 0.05, **P < 0.01 |  |  |  |  |
| ^a^Differences in population size are due to missing data at measurement. | |  |  |  |
| ^b^Whilst gender is included as predeployment, data on gender came from 2½-3y postdeployment. | | |  |  |
